# Supplementary material for: Heparin-based hydrogel scaffolding alters the transcriptomic profile and increases the chemoresistance of MDA-MB-231 triple-negative breast cancer cells
Source: Biomater Sci. 2020 Feb 13;8(10):2786–96. doi: 10.1039/c9bm01481k (PMC7497406; doi:10.1039/c9bm01481k)
Supplement: Supplementary file 2 [file BM-008-C9BM01481K-s002.zip › Supplementary File 4/HP-B hydrogel + EGF (s)vControl/Pathways/my_analysis.Gsea.1545201170987/HALLMARK_IL2_STAT5_SIGNALING.html]

Details for gene set HALLMARK\_IL2\_STAT5\_SIGNALING[GSEA]

|  || Dataset | expr.class.cls#HYDEGF\_versus\_CONTROL.class.cls#HYDEGF\_versus\_CONTROL\_repos |
| Phenotype | class.cls#HYDEGF\_versus\_CONTROL\_repos |
| Upregulated in class | CONTROL |
| GeneSet | HALLMARK\_IL2\_STAT5\_SIGNALING |
| Enrichment Score (ES) | -0.38832214 |
| Normalized Enrichment Score (NES) | -1.7934549 |
| Nominal p-value | 0.0 |
| FDR q-value | 0.0012652812 |
| FWER p-Value | 0.013 |
Table: GSEA Results Summary

  

Fig 1: Enrichment plot: HALLMARK\_IL2\_STAT5\_SIGNALING      
 Profile of the Running ES Score & Positions of GeneSet Members on the Rank Ordered List

  

| PROBE | DESCRIPTION (from dataset) | GENE SYMBOL | GENE\_TITLE | RANK IN GENE LIST | RANK METRIC SCORE | RUNNING ES | CORE ENRICHMENT || 1 | NOP2 | na |  |  | 279 | 2.413 | 0.0034 | No |
| 2 | AKAP2 | na |  |  | 295 | 2.392 | 0.0205 | No |
| 3 | GADD45B | na |  |  | 524 | 2.096 | 0.0241 | No |
| 4 | SLC1A5 | na |  |  | 679 | 1.922 | 0.0304 | No |
| 5 | PUS1 | na |  |  | 936 | 1.726 | 0.0299 | No |
| 6 | MYO1C | na |  |  | 1516 | 1.425 | 0.0101 | No |
| 7 | LRIG1 | na |  |  | 1869 | 1.304 | 0.0014 | No |
| 8 | ENO3 | na |  |  | 1899 | 1.295 | 0.0095 | No |
| 9 | HIPK2 | na |  |  | 2027 | 1.260 | 0.0123 | No |
| 10 | IL3RA | na |  |  | 2111 | 1.235 | 0.0171 | No |
| 11 | BATF3 | na |  |  | 2259 | 1.191 | 0.0183 | No |
| 12 | GPR83 | na |  |  | 2383 | 1.154 | 0.0205 | No |
| 13 | AGER | na |  |  | 2387 | 1.154 | 0.0290 | No |
| 14 | CDC6 | na |  |  | 2609 | 1.102 | 0.0256 | No |
| 15 | RABGAP1L | na |  |  | 2717 | 1.076 | 0.0280 | No |
| 16 | SCN9A | na |  |  | 2759 | 1.061 | 0.0338 | No |
| 17 | CCNE1 | na |  |  | 3012 | 1.001 | 0.0280 | No |
| 18 | APLP1 | na |  |  | 3214 | 0.956 | 0.0246 | No |
| 19 | NCS1 | na |  |  | 3343 | 0.934 | 0.0249 | No |
| 20 | FAM126B | na |  |  | 3933 | 0.824 | 0.0001 | No |
| 21 | ODC1 | na |  |  | 3955 | 0.820 | 0.0051 | No |
| 22 | SLC29A2 | na |  |  | 4230 | 0.777 | -0.0035 | No |
| 23 | MYC | na |  |  | 4314 | 0.764 | -0.0021 | No |
| 24 | MAFF | na |  |  | 4719 | 0.703 | -0.0181 | No |
| 25 | FAH | na |  |  | 4997 | 0.653 | -0.0277 | No |
| 26 | IKZF4 | na |  |  | 5129 | 0.631 | -0.0299 | No |
| 27 | LIF | na |  |  | 5147 | 0.629 | -0.0261 | No |
| 28 | TTC39B | na |  |  | 5210 | 0.619 | -0.0247 | No |
| 29 | CD44 | na |  |  | 5293 | 0.607 | -0.0245 | No |
| 30 | BCL2 | na |  |  | 5367 | 0.594 | -0.0239 | No |
| 31 | RNH1 | na |  |  | 5402 | 0.590 | -0.0213 | No |
| 32 | GLIPR2 | na |  |  | 6004 | 0.502 | -0.0491 | No |
| 33 | MAP3K8 | na |  |  | 6069 | 0.493 | -0.0487 | No |
| 34 | DENND5A | na |  |  | 6175 | 0.479 | -0.0507 | No |
| 35 | FGL2 | na |  |  | 6216 | 0.474 | -0.0492 | No |
| 36 | BATF | na |  |  | 6325 | 0.460 | -0.0515 | No |
| 37 | UCK2 | na |  |  | 6380 | 0.452 | -0.0509 | No |
| 38 | CYFIP1 | na |  |  | 6538 | 0.433 | -0.0559 | No |
| 39 | GALM | na |  |  | 6740 | 0.405 | -0.0635 | No |
| 40 | MAPKAPK2 | na |  |  | 6749 | 0.403 | -0.0609 | No |
| 41 | PNP | na |  |  | 6968 | 0.376 | -0.0695 | No |
| 42 | IGF1R | na |  |  | 6976 | 0.375 | -0.0671 | No |
| 43 | ALCAM | na |  |  | 7468 | 0.309 | -0.0906 | No |
| 44 | FURIN | na |  |  | 7555 | 0.298 | -0.0928 | No |
| 45 | S100A1 | na |  |  | 8257 | 0.215 | -0.1280 | No |
| 46 | SPRY4 | na |  |  | 8764 | 0.156 | -0.1534 | No |
| 47 | CCND3 | na |  |  | 8864 | 0.140 | -0.1576 | No |
| 48 | LCLAT1 | na |  |  | 9090 | 0.116 | -0.1685 | No |
| 49 | SLC39A8 | na |  |  | 9133 | 0.111 | -0.1699 | No |
| 50 | IRF6 | na |  |  | 9254 | 0.095 | -0.1755 | No |
| 51 | ST3GAL4 | na |  |  | 9289 | 0.090 | -0.1766 | No |
| 52 | PRKCH | na |  |  | 9546 | 0.069 | -0.1896 | No |
| 53 | TNFRSF1B | na |  |  | 9814 | 0.033 | -0.2033 | No |
| 54 | PTCH1 | na |  |  | 9867 | 0.027 | -0.2059 | No |
| 55 | CAPN3 | na |  |  | 9947 | 0.024 | -0.2098 | No |
| 56 | AHCY | na |  |  | 10060 | 0.011 | -0.2156 | No |
| 57 | CKAP4 | na |  |  | 10260 | -0.017 | -0.2260 | No |
| 58 | NFKBIZ | na |  |  | 10264 | -0.017 | -0.2260 | No |
| 59 | LRRC8C | na |  |  | 10567 | -0.046 | -0.2415 | No |
| 60 | UMPS | na |  |  | 10737 | -0.071 | -0.2498 | No |
| 61 | CD83 | na |  |  | 10860 | -0.087 | -0.2556 | No |
| 62 | MAP6 | na |  |  | 10915 | -0.096 | -0.2577 | No |
| 63 | PIM1 | na |  |  | 11181 | -0.119 | -0.2707 | No |
| 64 | ITGAE | na |  |  | 11198 | -0.122 | -0.2707 | No |
| 65 | RGS16 | na |  |  | 11341 | -0.139 | -0.2771 | No |
| 66 | SNX14 | na |  |  | 11411 | -0.147 | -0.2796 | No |
| 67 | SYT11 | na |  |  | 11416 | -0.148 | -0.2787 | No |
| 68 | PTRH2 | na |  |  | 11655 | -0.182 | -0.2898 | No |
| 69 | MYO1E | na |  |  | 11738 | -0.194 | -0.2927 | No |
| 70 | AMACR | na |  |  | 11754 | -0.196 | -0.2920 | No |
| 71 | CD81 | na |  |  | 11825 | -0.208 | -0.2942 | No |
| 72 | RHOB | na |  |  | 11865 | -0.214 | -0.2946 | No |
| 73 | PTGER2 | na |  |  | 12108 | -0.234 | -0.3056 | No |
| 74 | SOCS2 | na |  |  | 12143 | -0.236 | -0.3056 | No |
| 75 | PHTF2 | na |  |  | 12204 | -0.244 | -0.3069 | No |
| 76 | IL4R | na |  |  | 12312 | -0.263 | -0.3106 | No |
| 77 | SH3BGRL2 | na |  |  | 12615 | -0.301 | -0.3242 | No |
| 78 | F2RL2 | na |  |  | 12730 | -0.319 | -0.3278 | No |
| 79 | RHOH | na |  |  | 12866 | -0.335 | -0.3324 | No |
| 80 | GUCY1B3 | na |  |  | 12911 | -0.335 | -0.3322 | No |
| 81 | CAPG | na |  |  | 12979 | -0.337 | -0.3332 | No |
| 82 | POU2F1 | na |  |  | 13110 | -0.355 | -0.3373 | No |
| 83 | ARL4A | na |  |  | 13134 | -0.358 | -0.3359 | No |
| 84 | ETV4 | na |  |  | 13360 | -0.394 | -0.3447 | No |
| 85 | MXD1 | na |  |  | 13508 | -0.414 | -0.3494 | No |
| 86 | EOMES | na |  |  | 13536 | -0.419 | -0.3477 | No |
| 87 | CDKN1C | na |  |  | 13565 | -0.423 | -0.3460 | No |
| 88 | KLF6 | na |  |  | 13608 | -0.426 | -0.3450 | No |
| 89 | P2RX4 | na |  |  | 13816 | -0.461 | -0.3524 | No |
| 90 | COL6A1 | na |  |  | 13983 | -0.487 | -0.3575 | No |
| 91 | BCL2L1 | na |  |  | 14288 | -0.519 | -0.3696 | No |
| 92 | PLSCR1 | na |  |  | 14338 | -0.526 | -0.3682 | No |
| 93 | SNX9 | na |  |  | 14356 | -0.530 | -0.3652 | No |
| 94 | GPX4 | na |  |  | 14401 | -0.538 | -0.3635 | No |
| 95 | TNFRSF21 | na |  |  | 14534 | -0.561 | -0.3662 | No |
| 96 | TWSG1 | na |  |  | 14612 | -0.575 | -0.3659 | No |
| 97 | CCND2 | na |  |  | 14645 | -0.580 | -0.3633 | No |
| 98 | RRAGD | na |  |  | 14803 | -0.604 | -0.3670 | No |
| 99 | DCPS | na |  |  | 14828 | -0.608 | -0.3637 | No |
| 100 | ADAM19 | na |  |  | 14855 | -0.612 | -0.3605 | No |
| 101 | PDCD2L | na |  |  | 14916 | -0.621 | -0.3590 | No |
| 102 | CXCL10 | na |  |  | 14952 | -0.628 | -0.3562 | No |
| 103 | IKZF2 | na |  |  | 15094 | -0.651 | -0.3587 | No |
| 104 | ITGA6 | na |  |  | 15114 | -0.656 | -0.3548 | No |
| 105 | SYNGR2 | na |  |  | 15236 | -0.676 | -0.3561 | No |
| 106 | PRAF2 | na |  |  | 15850 | -0.791 | -0.3824 | Yes |
| 107 | NFIL3 | na |  |  | 15878 | -0.797 | -0.3779 | Yes |
| 108 | HK2 | na |  |  | 15926 | -0.806 | -0.3743 | Yes |
| 109 | CTSZ | na |  |  | 15985 | -0.815 | -0.3713 | Yes |
| 110 | TNFRSF9 | na |  |  | 16000 | -0.819 | -0.3659 | Yes |
| 111 | XBP1 | na |  |  | 16049 | -0.833 | -0.3622 | Yes |
| 112 | CSF2 | na |  |  | 16092 | -0.842 | -0.3581 | Yes |
| 113 | TIAM1 | na |  |  | 16222 | -0.879 | -0.3583 | Yes |
| 114 | BMPR2 | na |  |  | 16383 | -0.919 | -0.3598 | Yes |
| 115 | IGF2R | na |  |  | 16595 | -0.968 | -0.3637 | Yes |
| 116 | SWAP70 | na |  |  | 16696 | -0.999 | -0.3615 | Yes |
| 117 | SERPINB6 | na |  |  | 16763 | -1.014 | -0.3574 | Yes |
| 118 | BHLHE40 | na |  |  | 16798 | -1.027 | -0.3515 | Yes |
| 119 | TGM2 | na |  |  | 16812 | -1.030 | -0.3445 | Yes |
| 120 | NCOA3 | na |  |  | 16858 | -1.044 | -0.3390 | Yes |
| 121 | CDC42SE2 | na |  |  | 16875 | -1.047 | -0.3321 | Yes |
| 122 | IFITM3 | na |  |  | 16890 | -1.051 | -0.3249 | Yes |
| 123 | TRAF1 | na |  |  | 16954 | -1.073 | -0.3202 | Yes |
| 124 | SPRED2 | na |  |  | 17028 | -1.098 | -0.3159 | Yes |
| 125 | PRNP | na |  |  | 17039 | -1.101 | -0.3082 | Yes |
| 126 | TLR7 | na |  |  | 17186 | -1.138 | -0.3073 | Yes |
| 127 | CDCP1 | na |  |  | 17233 | -1.154 | -0.3011 | Yes |
| 128 | NRP1 | na |  |  | 17279 | -1.174 | -0.2947 | Yes |
| 129 | EMP1 | na |  |  | 17346 | -1.196 | -0.2892 | Yes |
| 130 | NT5E | na |  |  | 17372 | -1.205 | -0.2815 | Yes |
| 131 | CASP3 | na |  |  | 17375 | -1.207 | -0.2726 | Yes |
| 132 | ECM1 | na |  |  | 17433 | -1.227 | -0.2665 | Yes |
| 133 | SOCS1 | na |  |  | 17552 | -1.279 | -0.2631 | Yes |
| 134 | TNFSF10 | na |  |  | 17691 | -1.328 | -0.2604 | Yes |
| 135 | CST7 | na |  |  | 17828 | -1.401 | -0.2571 | Yes |
| 136 | PHLDA1 | na |  |  | 17992 | -1.481 | -0.2546 | Yes |
| 137 | DHRS3 | na |  |  | 18222 | -1.612 | -0.2546 | Yes |
| 138 | AHR | na |  |  | 18333 | -1.702 | -0.2476 | Yes |
| 139 | GSTO1 | na |  |  | 18377 | -1.735 | -0.2369 | Yes |
| 140 | ANXA4 | na |  |  | 18571 | -1.892 | -0.2329 | Yes |
| 141 | P4HA1 | na |  |  | 18626 | -1.958 | -0.2211 | Yes |
| 142 | WLS | na |  |  | 18628 | -1.960 | -0.2065 | Yes |
| 143 | BMP2 | na |  |  | 18629 | -1.962 | -0.1919 | Yes |
| 144 | SMPDL3A | na |  |  | 18641 | -1.976 | -0.1777 | Yes |
| 145 | ITGAV | na |  |  | 18729 | -2.091 | -0.1666 | Yes |
| 146 | CISH | na |  |  | 18778 | -2.156 | -0.1530 | Yes |
| 147 | LTB | na |  |  | 18802 | -2.196 | -0.1378 | Yes |
| 148 | NDRG1 | na |  |  | 18853 | -2.277 | -0.1235 | Yes |
| 149 | PLIN2 | na |  |  | 18894 | -2.342 | -0.1081 | Yes |
| 150 | IFNGR1 | na |  |  | 18912 | -2.376 | -0.0912 | Yes |
| 151 | SLC2A3 | na |  |  | 19022 | -2.648 | -0.0771 | Yes |
| 152 | GBP4 | na |  |  | 19050 | -2.740 | -0.0581 | Yes |
| 153 | GABARAPL1 | na |  |  | 19071 | -2.800 | -0.0382 | Yes |
| 154 | ENPP1 | na |  |  | 19105 | -2.928 | -0.0181 | Yes |
| 155 | RORA | na |  |  | 19128 | -3.064 | 0.0037 | Yes |
Table: GSEA details [plain text format]

  

Fig 2: HALLMARK\_IL2\_STAT5\_SIGNALING      
 Blue-Pink O' Gram in the Space of the Analyzed GeneSet

  

Fig 3: HALLMARK\_IL2\_STAT5\_SIGNALING: Random ES distribution      
 Gene set null distribution of ES for **HALLMARK\_IL2\_STAT5\_SIGNALING**
